# Response of Endophytic Microbial Communities and Quality of *Salvia miltiorrhiza* to Fertilization Treatments

**DOI:** 10.3390/microorganisms13061429

**Published:** 2025-06-19

**Authors:** Wenjing Chen, Wanyun Li, Yangyang Pan, Xin Zheng, Xinxin Fu, Menghui Wang, Wenyi Shi, Zhenzhou Wang, Xueli He, Chao He, Xianen Li

**Affiliations:** 1 Institute of Medicinal Plant Development, Chinese Academy of Medical Sciences & Peking Union Medical College, Beijing 100193, China; chenwenjing09@126.com (W.C.); liwanyun0623@126.com (W.L.); 2 School of Life Sciences, Hebei University, Baoding 071002, China; pyyjy15033247072@126.com (Y.P.); zx1179006820@163.com (X.Z.); 15102606871@163.com (X.F.); wangmenghui0225@163.com (M.W.); shiwenyi9475@163.com (W.S.); dddsxwzz@126.com (Z.W.); xlh3615@126.com (X.H.)

**Keywords:** *Salvia miltiorrhiza*, fertilization treatment, endophytic microbial community

## Abstract

*Salvia miltiorrhiza* is a traditional herbal remedy for cardiovascular diseases and is in high demand in the market. Excessive chemical fertilizer application, resulting from unscientific fertilization practices, reduced the tanshinone content in *S. miltiorrhiza* roots. This study investigated how different fertilization types alter the endophytic microbial community composition of *S. miltiorrhiza* through field experiments, aiming to understand how fertilization affects its medicinal quality. The results showed that root fertilizers (F1) significantly increased root biomass and tanshinone I content, whereas foliar fertilizers (F2) increased tanshinone IIA content. High-throughput sequencing further revealed that F2 treatment significantly decreased the Shannon index of endophytic bacteria while significantly increasing the Shannon index of endophytic fungi. Co-occurrence network analysis revealed that fertilization significantly altered fungal community complexity and modularity, with F1 increasing network nodes and edges. Variance partitioning analysis indicated fungal diversity more strongly influenced medicinal compound levels under F2 and a combination of both (F3) than bacterial diversity. *Septoria* and *Gibberella* were positively correlated with tanshinone I and cryptotanshinone content under F2 treatment, respectively. Notably, the unique strains were isolated from different fertilization treatments for subsequent bacterial fertilizer development. These findings elucidate microbial responses to fertilization, guiding optimized cultivation for improved *S. miltiorrhiza* quality.

## 1. Introduction

*Salvia miltiorrhiza* is a bulk-type medicinal herb belonging to the Labiatae family, primarily used for its dried root and rhizomes. Its primary medicinal ingredients include fat-soluble (tanshinone) compounds and water-soluble (salvianolic acid B, rosmarinic acid, etc.) compounds [1]. Studies indicate that tanshinone exhibits pharmacological effects such as promoting blood circulation, resolving stasis [2], antioxidant activity [3], and anti-inflammatory properties [4]. *S. miltiorrhiza* has been used to treat cardiovascular illnesses [5]. The demands of the *S. miltiorrhiza* industry can be satisfied via artificial cultivation [6]. In the cultivation process of *S. miltiorrhiza*, fertilizer is currently a widespread way to increase production. Chemical fertilizers are the most widely utilized fertilizers [7]. Excessive fertilization has been found in studies to reduce plant biomass and lower quality. Meanwhile, excessive fertilizer application induces soil salinization and nutrient imbalances, which consequently compromise plant growth and medicinal quality [8]. Therefore, there is an urgent need to guide the *S. miltiorrhiza* industry to optimize fertilizer use to improve the quality of artificial cultivation of *S. miltiorrhiza*.

Foliar fertilization is a method of delivering nutrients to plants by spraying liquid fertilizer onto the surface of the leaves [9]. With its rapid nutrient uptake, high efficacy, low consumption, and reduced environmental contamination, foliar fertilizer is a valuable addition to soil fertilization and an efficient agricultural technique [10]. More studies have been conducted on how foliar fertilization can improve the quality of plants [11,12]. In addition, some researchers have found that foliar fertilization can also change the microbial composition. Chen et al. [13] found that foliar application of small peptides decreased the bacterial diversity of the interleaf microbiome of tea trees and increased the diversity of fungi. Wang et al. [14] reported that foliar application of liquid bio-fertilizer AA9 significantly reduced the Shannon index of bacterial communities (*p* < 0.05) compared to the untreated control, while there was no significant change in the fungal Shannon index. In contrast, there are fewer studies on how foliar fertilization affects the microbial diversity of *S. miltiorrhiza*. Gong et al. [15] found that the number of bacterial and fungal OTUs in different ecological niches of *S. miltiorrhiza* decreased under foliar fertilization but increased the accumulation of medicinal constituent content of *S. miltiorrhiza*. In addition, microbial communities reside not only on the plant surface but also in surrounding soil and internal tissues [16].

Endophytic microflora are microbial taxa that do not cause visible disease symptoms in plant tissues [17]. They mainly include endophytic bacteria, endophytic fungi, and actinomycetes [5]. Endophyte–plant symbiotic interactions have been extensively studied, with endophytes increasing the plant uptake and utilization of soil nutrients through mineral solubilization and synthesis of chelated iron [18]. Yuan et al. [19] found that soil nutrients were the main determinants of the structural changes in the bacterial community, which altered the inter-root microbial community of sugarcane through redundancy analysis. In addition, many studies have shown that endophytes can produce many bioactive secondary metabolites, which are novel natural product resources [20]. In contrast to the extensive research on endophytic fungal isolation and application in *S. miltiorrhiza*, investigations examining the influence of differential fertilizer applications on its endophytic microbial community composition remain notably scarce. Li et al. [21] inoculated *S. miltiorrhiza* with nine strains of endophytic fungi isolated from the *S. miltiorrhiza* root system to obtain growth-promoting strains of beneficial fungi. Zhai et al. [22] reported that the endophytic fungus *Chaetomium globosum* D38, which was isolated from the root system of *S. miltiorrhiza*, could promote the growth of *S. miltiorrhiza* and the content of medicinal constituents. Lv et al. [23] investigated the mechanism of endophytic fungi regulating the tanshinone biosynthesis pathway by preparing the endophytic fungus *Penicillium speckii* DF33 inducer of *S. miltiorrhiza*. The above study demonstrated that the study of endophytic fungi of *S. miltiorrhiza* provides an essential foundation for the quality improvement process of *S. miltiorrhiza*.

In this study, we investigated the effects of various fertilization treatments—control, root fertilizer, foliar fertilizer, and a combination of root and foliar fertilizers—on the endophytic microbial community, plant growth, and medicinal components of *S. miltiorrhiza* through a traditional field cultivation experiment. We employed Illumina MiSeq high-throughput sequencing (HTS) technology to analyze changes in microbial abundance, diversity, and community composition across different ecological niches of *S. miltiorrhiza* under the various fertilization treatments [15]. The results from high-throughput sequencing were categorized into culturable and culture-independent microorganisms [24], utilizing culture methods for isolation and purification [25] to further explore the composition of endophytic microbial communities in *S. miltiorrhiza* under the different fertilization treatments. We proposed the following hypotheses: (1) fertilizer treatments affect the growth and medicinal components of *S. miltiorrhiza*, and (2) fertilizer treatments alter the composition and diversity of endophytic microorganisms. The objectives of this study were to (1) characterize the endophytic bacteria and fungi associated with *S. miltiorrhiza,* and (2) determine fertilization-induced variations in microbial community structure, plant growth performance, and medicinal ingredients. Understanding the host–microbial interactions of *S. miltiorrhiza* can provide valuable theoretical insights for the development and utilization of endophytic microorganisms associated with *S. miltiorrhiza* and contribute to sustainable practices in medicinal plant cultivation.

## 2. Materials and Methods

### 2.1. Study Site

The field experiment was conducted in Yaozhou district, Tongchuan city, Shanxi province (108°34′~109°06′ E, 34°48′~35°19′ N). The study area has a continental climate, with an average annual sunshine duration of 2356.6 h, average annual precipitation of 554.5 mm, and average annual temperature ranging from 8.4 °C to 12.3 °C. The soil physicochemical properties were as follows: soil organic matter 4.454 × 10^4^ mg/kg, total nitrogen 170.247 mg/kg, available phosphorus 10.214 mg/kg, and available potassium 58.078 mg/kg.

### 2.2. Experimental Design

The field experiment employed a randomized block design comprising four fertilization treatments: (1) an untreated control, (2) root application alone, (3) foliar application alone, and (4) combined root + foliar application, with each plot covering an area of 16 m^2^ (4 × 4 m). Three replications were set for each fertilization treatment, resulting in a total of 12 sample plots (4 treatments × 3 replications). A protective area was established around the experimental plots. The *S. miltiorrhiza* used in this experiment were seedlings of the Ludan No. 1 variety, with a root length of approximately 15 cm and a root diameter of 0.5 cm or more, and free from pests, diseases, and injuries. The root fertilizer used was a STANLEY nitrogen–phosphorus–potassium complex fertilizer, applied at a rate of 37.5 kg per acre. The foliar fertilizer used was STANLEY potassium phosphate foliar fertilizer, applied at a rate of 50–60 g per acre. The root fertilizer was applied to the soil before transplanting the seedlings, while the foliar fertilizer was sprayed three times at 15-day intervals, starting from the vigorous growth period of the seedlings. Each sample plot was bordered by a 1 m wide, 30 cm high ridge, and a 25 cm wide drainage ditch. Two rows of *S. miltiorrhiza* were planted per ridge, with a spacing of 0.3 × 0.25 m between plants, and the rows were spaced 0.3 × 0.25 m apart.

### 2.3. Sample Collection

The experiment was undertaken in March 2022, with samples collected on 25 October 2022. Fifteen healthy plants and their rhizosphere soil were randomly collected from each plot. The tissues of *S. miltiorrhiza* were collected with a sterile knife. Samples of plants were kept in a refrigerator at -80 °C for high-throughput sequencing analysis, while some were used for endophytic fungus isolation. A rhizosphere soil sample was taken with a brush and sieved (<2 mm screen) to remove apoplastic debris, stones, and coarse roots. Rhizosphere soil samples for enzymatic analysis were kept in a freezer at 4 °C, while the remainder were allowed to dry naturally to determine soil physicochemical parameters.

### 2.4. Determination of Medicinal Ingredient Content

The samples of *S. miltiorrhiza* were dried at 80 °C to determine the plant’s aboveground and root biomass. The Chinese Pharmacopoeia was used to determine the medicinal components of *S. miltiorrhiza*, and the following chromatographic conditions were used: Octadecylsilane-bonded silica gel serves as the packing material, and acetonitrile and a solution of 0.05% phosphoric acid serve as the mobile phases A and B, respectively. Tanshinone I, tanshinone IIA, and cryptotanshinone were the fat-soluble components that were identified by weighing 0.5 g of powder in 50 mL of methanol after the dried roots were ground through a screen. Elution is carried out according to the conditions in the table (Supplementary Table S1). The flow rate was 1 mL/1 min, and the detection wavelength was 270 nm. We weighed 0.15 g of roots and fixed them to 50 mL with an 80% methanol solution; the flow rate was 1 mL/min; and a detection wavelength of 286 nm allowed us to identify the water-soluble components (salvianolic acid B and rosmarinic acid).

### 2.5. Extraction of DNA and Illumina MiSeq Sequencing

A 5 g sample of *S. miltiorrhiza* was placed in centrifuge tubes with 0.1 μM potassium phosphate buffer and sonicated at 40 kHz for 1 min, followed by shaking at 200 rpm for 3 min, and this procedure was repeated 2–3 times [26]. The samples were surface-sterilized sequentially with alcohol (75%, 4 min) and sodium hypochlorite (5%, 2 min) and washed 3 times with sterile water. Each treatment was repeated 3 times. The genomic DNA of *S. miltiorrhiza* samples was extracted using the FastDNA^®^ Soil DNA Spin Kit. The 16S rDNA target region of the ribosomal RNA gene and the internal transcribed spacer (ITS) target region were amplified via PCR, as follows: 95 °C for 3 min; denaturation at 95 °C for 30 s; primer annealing at 55 °C for 30 s; extension at 72 °C for 45 s for a total of 27 cycles; finally, extension at 72 °C for 10 min. The primer sequences of the 16S rDNA V3-V4 region were 338 F, CTCCTACGGGGGAGGCAGCAG, and 806 R, GGACTACHVGGGGTWTCTAAT [15]; those of the fungal ITS rRNA region were ITS1F (5′-CTTGGTCATTTAGAGGAAGTAA-3′) and ITS2-2043R (5′-GCTGCGTTCTTCATCGATGC-3ʹ) [27]. All PCR reactions were carried out with 15 µL of Phusion^®^ High-Fidelity PCR Master Mix (New England Biolabs, Ipswich, MA, USA), 0.2 µM of forward and reverse primers, and about 10 ng of template DNA. The PCR products were subsequently purified, followed by quality and quantification tests using 2% agarose electrophoresis and QC-agarose electrophoresis, respectively. Agarose electrophoresis and Quanti Fluor™-ST (Promega, Madison, WI, USA), respectively. Finally, a 2*300 bp library was constructed. Sequencing was performed using the Illumina MiSeq platform (PE300, Illumina, San Diego, CA, USA). The raw reads were deposited in the National Center for Biotechnology Information (NCBI) Sequence Read Archive (SRA) database (Accession Number: PRJNA1122354 and PRJNA1122931).

After obtaining the sequencing data, the raw data were first spliced and filtered to obtain valid data. Then, based on the valid data, noise reduction was performed by DADA2 or deblur (DADA2 was used by default) to obtain the final ASVs [28]. The final ASVs were obtained using the RDP classifier (version 2.11) [29] of QIIME2 with the Silva 16S rRNA gene database (version 138.1) and Unite (version 8.2) databases [30] as the basis for species annotation and abundance information for 16S rRNA and ITS representative ASV sequences.

### 2.6. Bioinformatic Analysis

The DNA sequences of the isolated endophytic fungus were sheared using Chromas (version 2.6.6) and examined using the BLAST tool (https://blast.ncbi.nlm.nih.gov/Blast.cgi, accessed on March 2025) of the NCBI to screen out model strains with high homology, and the ML phylogenetic tree was built with MEGA 7.0.26 software. The Shannon diversity index was calculated using QIIME [15] (Version QIIME2-202202). Non-metric multidimensional analysis (NMDS) was then performed using the ade4 package and the ggplot2 package from the R project (http://www.R-project.org, accessed on 16 April 2025). Phylum-level symbiotic network analyses of endophytic fungal and endophytic bacterial communities from different fertilization treatments were performed using R Studio 4.1.2 and Gephi software (version 0.9.2). The endophytic fungal and bacterial communities were analyzed using FUNGuild [31] (version 1.0) to infer fungal function and use PICRUSt2 [32] (v2.3.0) to predict bacterial function.

### 2.7. Soil Physicochemical Properties

A precise pH meter was used to determine the pH of soil (soil–water volume ratio of 1:2.5). The coking mass method was used to determine organic matter (SOM); soil samples were placed in an oven at 110 °C for 2 h before being completely combusted in a muffle furnace at 550 °C for 4 h [33]. The levels of ammoniacal nitrogen (NH_4_) and nitrate nitrogen (NO_3_) were determined with a completely automated chemical element analyzer (SmartChem 200, Alliance, Paris, France) [34]. The modified Tabatabai method determined alkaline phosphatase (ALP) activity [35]. Urease activity (URE) was determined by Hoffmann and Teicher’s colorimetric method [36]. Available phosphorus (AP) was determined by the sodium bicarbonate leaching-molybdenum-antimony colorimetric method [37]. Available potassium (AK) was determined by the sodium tetraphenyl boronate-turbidimetric method [38], total phosphorus (TP) was determined by the molybdenum-vanadium blue colorimetric method [39], total nitrogen (TN) was determined by the Kjeldahl nitrogen determination method [40], and sucrase (SC) was measured by the colorimetric method of 3,5-dinitrosalicylic acid [41]. Sachdeva’s method determined nitrate reductase (NR) activity [42].

### 2.8. Isolation of Endophytic Fungi and Identification

The tissues of *S. miltiorrhiza* were collected and surface sterilized (75% alcohol for 2 min, sterile water rinsing for 2–3 times, 5% NaClO disinfection for 4 min, sterile water rinsing for 3 times) to remove surface microorganisms. Sterilized tissue samples were aseptically transferred onto individual PDA plates, then incubated upside-down at 28 °C in the dark using a constant temperature incubator. A purification culture was carried out following the formation of colonies surrounding the tissues. The strain morphology was photographed, and the micromorphology of the isolated strains was examined using a BS53 microscope.

We extracted 20 mg of fresh mycelium from purified PDA plates in 1.5 mL centrifuge tubes. Mycelial DNA was extracted using the alkaline lysis method [43]. Primers ITS4 (5′-TCCTCCGCTTATTGATATATATGC-3′) and ITS5 (5′-GGAAGTAAAAGTCGTAACAAGG3′) were used to amplify the ITS region. A 25 μL PCR reaction system was used: 3 μL of fungal DNA template, 1 μL of ITS4 primer, 1 μL of ITS5 primer, and 20 μL of 2 × Es Taq Master Mix. PCR cycling was carried out on a LifeECOTM system (BIOER, Hangzhou, China), and the reaction program was as follows: initial denaturation at 94 °C for 5 min; denaturation at 94 °C for 1 min; primer annealing at 55 °C for 1 min; extension at 72 °C for 1 min; a total of 35 cycles; and finally, extension at 72 °C for 10 min [44]. The PCR products were purified and sent to General Biologicals Ltd. (Taiwan) for genome sequencing. The aligned and edited sequences were deposited in the NCBI database with collection numbers OR343631-OR343647, OR343649, OR343654-OR343658, OR363183, OR363179, OR343652, and OR363180.

### 2.9. Statistical Analysis

The rhizosphere soil physicochemical properties of *S. miltiorrhiza* were processed using Excel 2019 software and expressed as the mean ± standard error (3 replications). One-way analysis of variance (ANOVA) was performed using IBM SPSS Statistics (version 25.0) software, and effects were considered significant if *p* < 0.05. The box plots were plotted using Origin 24.0. Variance partitioning analysis (VPA) was conducted with the ‘vegan’ package (RStudio 4.3.3) to disentangle the effects of endophytic communities on plant growth parameters and medicinal ingredient contents in *S. miltiorrhiza*.

## 3. Results

### 3.1. Determination of Growth Indexes and Medicinal Ingredient Contents of S. miltiorrhiza

**Figure 1:**
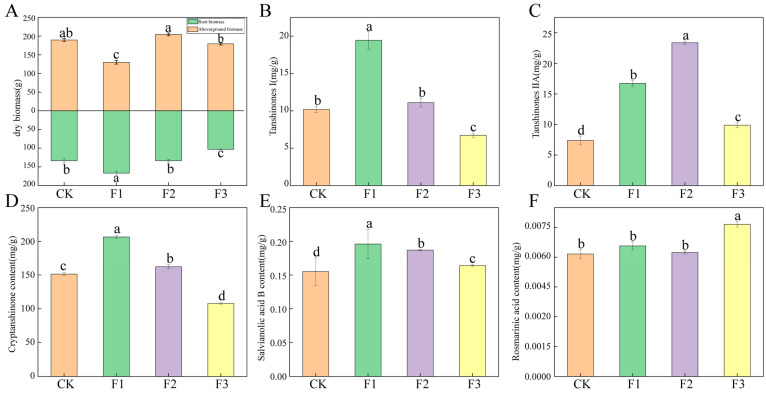
Effects of different fertilization treatments on growth indexes and medicinal ingredient content of *S. miltiorrhiza*. (**A**): dry biomass; (**B**): tanshinone I content; (**C**): tanshinone IIA content; (**D**): cryptotanshinone content; (**E**): salvianolic acid B content; (**F**): rosmarinic acid content. Different letters above the error line indicate a significant difference (*p* < 0.05). The same below.

Compared to CK treatment, the F1 treatment significantly increased the root dry weight of *S. miltiorrhiza*, while the root dry weight was significantly reduced under the F3 treatment. Furthermore, the aboveground dry biomass of *S. miltiorrhiza* was significantly higher in the F2 treatment, whereas it was significantly lower in the F1 treatment (Figure 1A). Different fertilization treatments significantly enhanced the content of salvianolic acid B in the root system of *S. miltiorrhiza* (Figure 1E). However, they had no significant effect on the content of rosmarinic acid (Figure 1F). Notably, the F1 treatment significantly increased the content of salvianolic acid B, tanshinone I, and cryptotanshinone in the roots, while the content of tanshinone I and cryptotanshinone was significantly lower under the F3 treatment compared to CK (Figure 1B,D). Additionally, both the F1 and F2 treatments significantly increased the content of tanshinone IIA in the roots (Figure 1C). When applied individually, both root and foliar fertilizers significantly enhanced the content of tanshinones, with the root fertilizer demonstrating the most effective results; however, co-application reduced root weight compared to individual treatments.

### 3.2. Changes in the Composition of the Endophyte Community of S. miltiorrhiza

**Figure 2:**
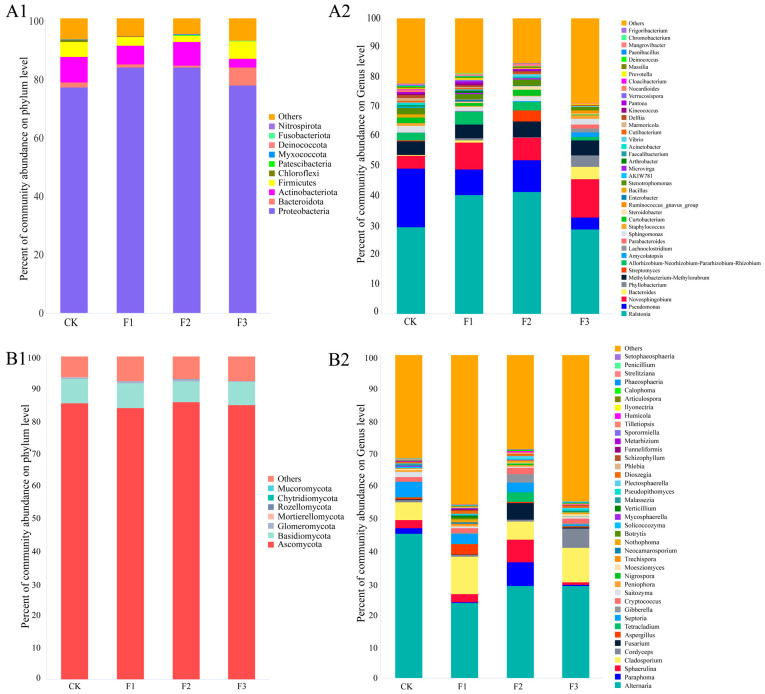
Changes in microbial community composition of *S. miltiorrhiza* under different fertilization treatments. (**A**) Endophytic bacterial community. (**B**) Endophytic fungal community. 1–2, species composition at the phylum level, and species composition at the genus level of different fertilization treatments on growth indexes and medicinal ingredient content of *S. miltiorrhiza*.

The exponential dilution curves of the Goods’ coverage for all samples at the OTU level were generally smooth, indicating that the sequencing depth met the requirements for subsequent analysis (Supplementary Figure S1). The 1243 bacterial OTUs obtained by sequencing belonged to 22 phyla, 38 classes, 100 orders, 171 families, and 337 genera. At the phylum level, the highest proportion of Proteobacteria was found in *S. miltiorrhiza* under different fertilization treatments (Figure 2(A1)). Notably, the proportion of Proteobacteria increased under all treatments compared to the control, with the F1 and F2 treatments showing the greatest increase. Further analyzing the top 40 abundance of genera, *Ralstonia* had the highest percentage under different fertilization treatments with 29.46%, 40.23%, 41.33%, and 28.70%, respectively (Figure 2(A2)). Compared to the control, the fertilizer treatments all increased the proportion of *Novosphingobium*. The F3 treatment increased *Phyllobacterium* and *Bacteroides*, while *Ralstonia* decreased.

Different fertilization treatments influenced the relative abundance of endophytic microorganisms of *S. miltiorrhiza*. A total of 1933 fungal OTUs were identified, belonging to 9 phyla, 29 classes, 61 orders, 135 families, and 235 genera. At the phylum level, Ascomycetes exhibited the highest relative abundance, exceeding 80% across the various fertilization treatments (Figure 2(B1)). In comparison to the F1 and F2 treatments, the abundance of Mortierellomycota and Rozellomycota was absent under the F3 treatment, while the abundance of Mucoromycota increased. Further analysis of the top 40 genera by abundance across different fertilizer treatments revealed that *Alternaria* was the dominant genus. The proportions of *Alternaria* found in the CK, F1, F2, and F3 treatments were 44.72%, 23.25%, 28.61%, and 28.60%, respectively, indicating that fertilizer application may have reduced the overall proportion of *Alternaria* (Figure 2(B2)). Additionally, it is noteworthy that fertilization treatments also decreased the proportion of *Aspergillus*. Compared to the CK treatment, the F2 treatment increased the abundance of *Paraphoma*, *Sphaerulina*, and *Cladosporium*, while both the F1 and F3 treatments resulted in a decrease in these genera.

### 3.3. Changes in the OTU Composition of the Endophyte Community of S. miltiorrhiza

**Figure 3:**
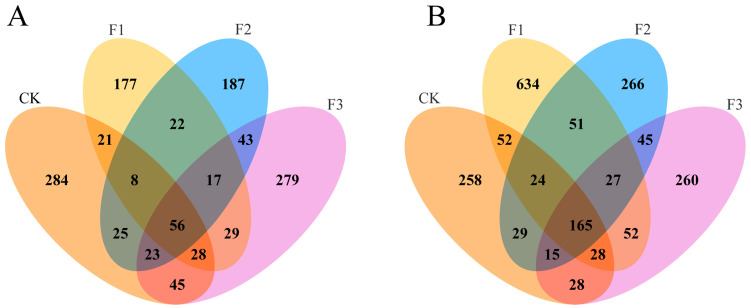
The distribution of endophytic microbial OTUs of *S. miltiorrhiza* under different treatments. (**A**) Endophytic bacteria; (**B**) endophytic fungi.

Different fertilization treatments altered the OTU composition of endophytic microorganisms in *S. miltiorrhiza* tissues. Venn diagrams further illustrated these changes (Figure 3). Under CK, F1, F2, and F3 treatments, there were 284, 177, 187, and 279 unique bacterial OTUs, respectively (Figure 3A). The application of root fertilizer, foliar fertilizer, or their combination reduced bacterial OTUs in *S. miltiorrhiza* tissues. Additionally, 56 bacterial OTUs were shared across treatments. Changes in fungal OTU counts contrasted with those in bacteria: CK, F1, F2, and F3 treatments yielded 258, 634, 266, and 260 fungal OTUs, respectively (Figure 3B). Among all treatments, the highest number of OTUs was observed in the F1 treatment, which suggests that root fertilization might enhance fungal richness. A total of 165 fungal OTUs were shared across treatments. Notably, endophytic microbial communities across fertilization treatments shared strains, with F2–F3 sharing significantly more strains than F1–F2 or F1–F3.

### 3.4. Changes in the Endophyte Community Diversity of S. miltiorrhiza

**Figure 4:**
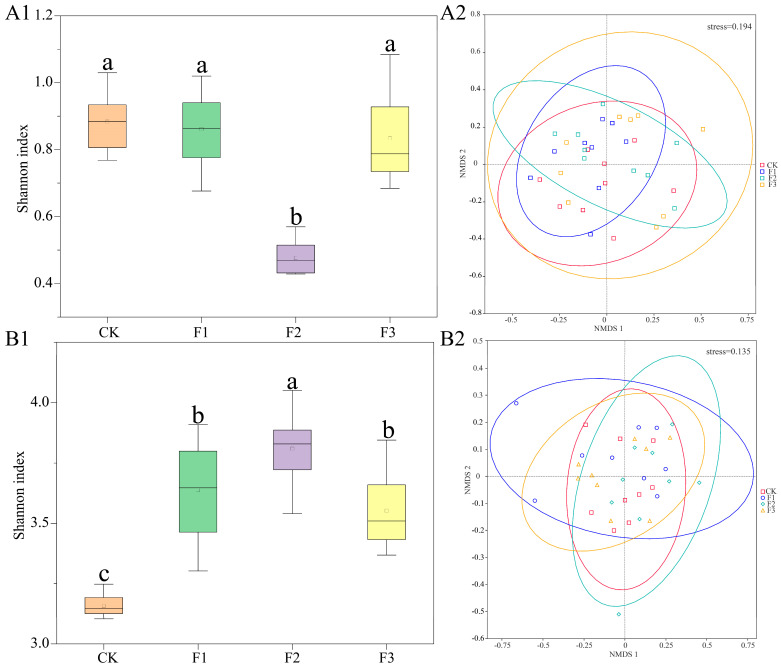
Changes in diversity of *S. miltiorrhiza* under different fertilization treatments. (**A**) Endophytic bacteria. (**B**) Endophytic fungi. 1 Shannon index (identical superscript letters indicate a nonsignificant difference, while distinct superscript letters denote statistical significance). 2 Non-metric multidimensional scaling (NMDS) analysis.

Different fertilization treatments influenced the diversity changes in endophytic microorganisms in *S. miltiorrhiza* (Figure 4). While none of the fertilization treatments increased the bacterial Shannon index relative to the control, the F2 treatment demonstrated a significant decrease. The endophytic fungal community changes in *S. miltiorrhiza* exhibited the opposite trend to the bacterial community under different fertilization treatments. All fertilization treatments significantly elevated the Shannon diversity index of endophytic fungal communities (*p* < 0.05). Notably, compared to the control, the endophytic fungal community’s Shannon index demonstrated a significant 20.6% increase under the F2 treatment, with the F1 and F3 treatments showing comparatively lower increases of 15.2% and 12.7%, respectively.

NMDS analysis based on the Bray–Curtis distance showed no significant separation of endophytic bacterial or fungal community structures in *S. miltiorrhiza* across fertilizer treatments (Figure 4(A2,B2)). While subtle differences in community composition were observed among treatments, these variations were not statistically significant, indicating limited fertilizer-type effects on microbial community assembly.

### 3.5. Co-Occurrence Network Analysis

**Figure 5:**
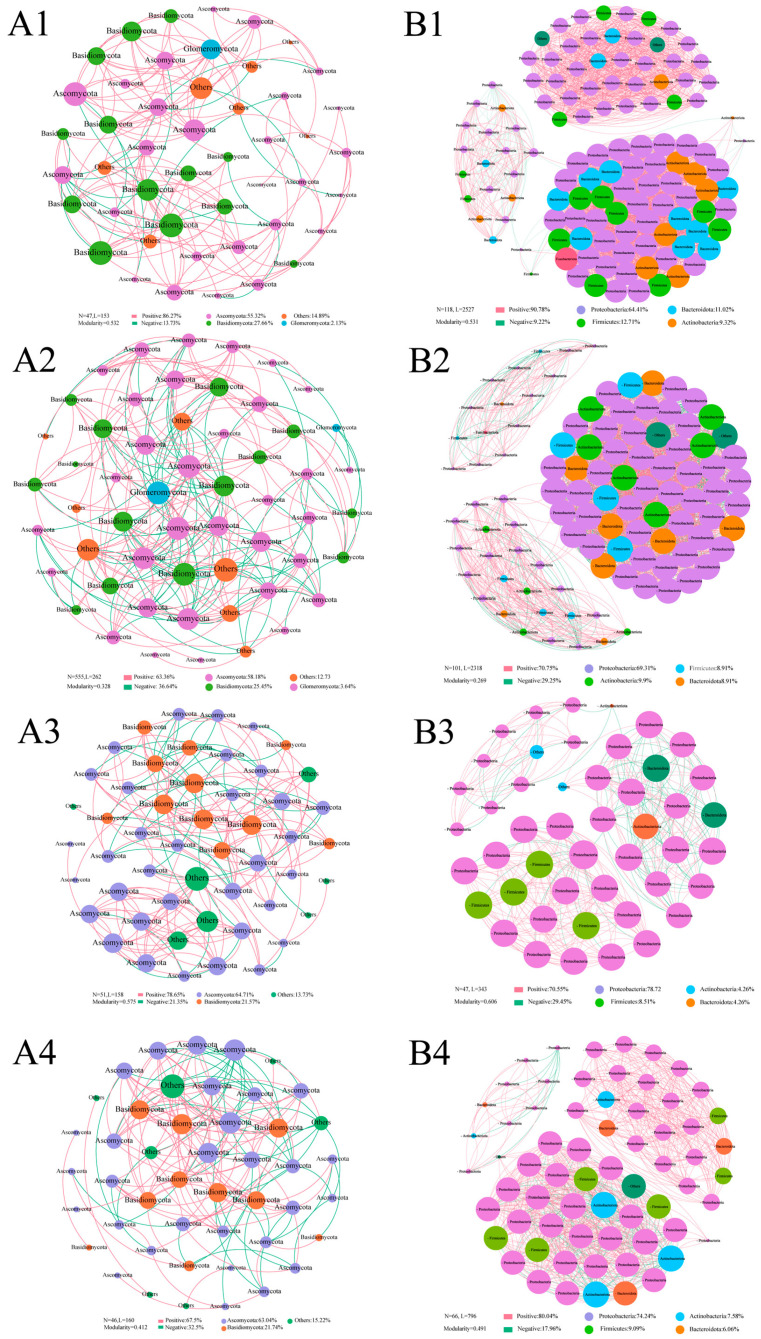
Co-occurrence network analysis based on the *S. miltiorrhiza* phylum level. (**A**) Endophytic fungi; (**B**) endophytic bacteria. 1–4 CK, F1, F2, and F3 treatments. Nodes represent OTUs, node color is used to distinguish different phyla, and OTU abundance is indicated by node size. The connecting line indicates a significant interaction between OTUs, the green color is a positive correlation, the red color is a negative correlation, and the thickness of the connecting line represents the correlation coefficient between nodes.

The OTU data were used to construct co-occurrence networks representing endophytic bacterial and fungal community structures in *S. miltiorrhiza* under different fertilizer treatments (Figure 5). In fungal communities, both node and edge counts were higher in F1 and F2 treatments than in the control (*p* < 0.05). In contrast, the F3 treatment only showed increased edge numbers relative to the control (Figure 5(A4)), with no significant node number increase (*p* > 0.1), potentially indicating a fertilizer mixing effect. Modularity indices were 0.532 (CK), 0.328 (F1), 0.575 (F2), and 0.472 (F3), with positive correlation proportions of 86.3%, 63.4%, 78.7%, and 67.5%, respectively (Figure 5(A1–A4)). Both metrics decreased under the F1 and F3 treatments but increased under the F2 treatment. Higher modularity indices (>0.5) generally indicate more stable ecological networks. Ascomycota and Basidiomycota constituted the dominant fungal phyla across all treatments.

In the bacterial community, the number of nodes was reduced under fertilization treatment, decreasing to 101 (F1), 47 (F2), and 66 (F3) compared to the control (118 nodes) (Figure 5B). The number of edges also decreased across all fertilization treatments. Unlike the fungal community, the modularity index of the bacterial community decreased in both F1 and F3 treatments but increased under F2 (Figure 5(B2–B4)). The modularity indices were 0.531 (CK), 0.269 (F1), 0.606 (F2), and 0.491 (F3), respectively. The higher modularity in F2 suggests greater community stability. Additionally, Proteobacteria and Firmicutes were the dominant bacterial phyla in all treatments. Compared to the control, the abundance of Proteobacteria increased, while that of Firmicutes decreased under all fertilizer treatments, with a significant increase in Proteobacteria observed under F3 (Figure 5(B4)). These results indicate that fertilization altered the bacterial community structure.

### 3.6. Functional Prediction

**Figure 6:**
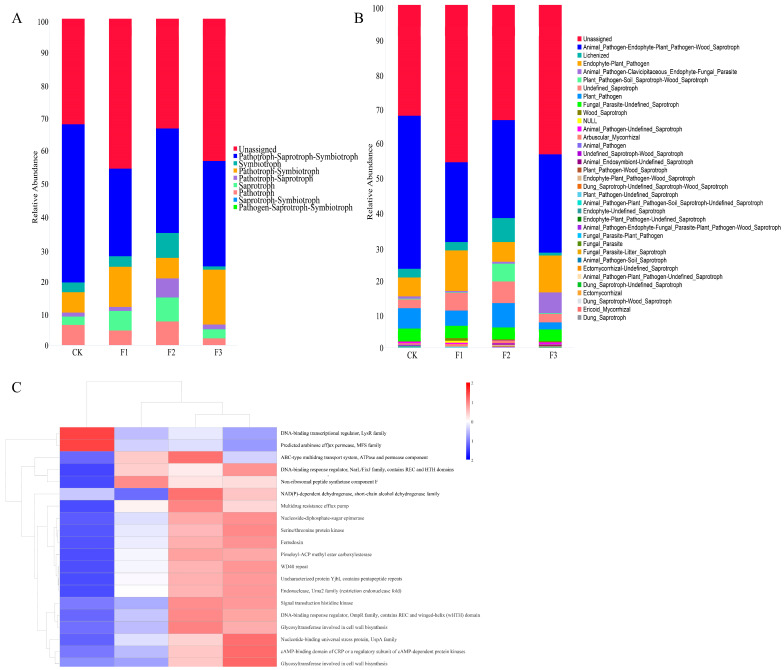
Functional prediction of endophytes in different ecological niches of *S. miltiorrhiza* under different fertilization treatments. (**A**,**B**) Histograms of relative abundance and functional classification of FunGuild functionally annotated endophytic fungi, respectively. (**C**) Heatmap of predicted functional abundance clustering (based on PICRUSt2 database) for endophytic bacterial communities in *S. miltiorrhiza* under different fertilization treatments.

Functional guild prediction of the endophytic fungal community in *S. miltiorrhiza*, conducted using the FUNGuild database, revealed significant fertilization-induced shifts in microbial functional profiles (Figure 6A). Fungal taxa were classified into nine trophic modes based on resource utilization strategies: (1) pathogen–saprotroph–symbiotroph, (2) saprotroph–symbiotroph, (3) pathotroph, (4) saprotroph, (5) pathotroph–saprotroph, (6) pathogen–symbiotroph, (7) symbiotroph, (8) pathotroph–saprotroph–symbiotroph, and (9) unclassified fungi. The pathotroph–saprotroph–symbiotroph guild represented the dominant functional group across treatments, although all guilds exhibited reductions in relative abundance compared to the CK control. These eight trophic modes encompassed 35 specific functional subcategories (Figure 6B). Among these, the animal pathogen–endophyte–plant pathogen–wood saprotroph maintained a predominance under all fertilizer treatments. Notably, fertilizer applications increased the abundance of two functional subcategories: animal endosymbiont–undefined saprotroph and animal pathogen–endophyte–fungal parasite–plant pathogen–wood saprotroph. Conversely, the animal pathogen–clavicipitaceous endophyte–fungal parasite group declined. These findings demonstrate that fertilization treatments selectively modulate microbial functional potential in *S. miltiorrhiza*.

Functional annotation of the endophytic bacterial community in *S. miltiorrhiza* was performed using PICRUSt2. It is noteworthy that protein functions were higher under the F3 treatment than under the other treatments. For example, protein functions of the DNA-binding response regulator as well as the predicted arabinose efflux permease were significantly enhanced. These functions play important roles in plant growth and development and the stress response (Figure 6C).

### 3.7. Effect of Different Fertilizer Treatments on Soil Parameters

**Figure 7:**
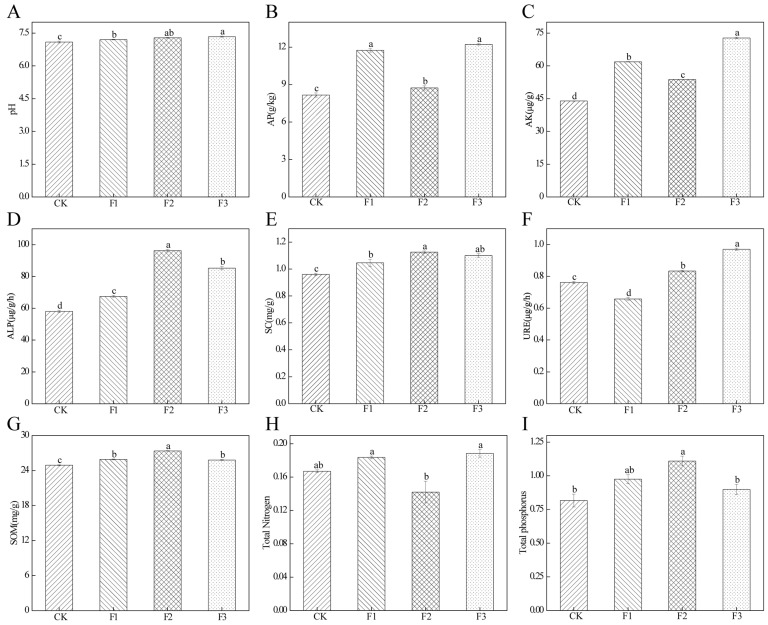
Effect of different fertilizer treatments on soil factors. (**A**) Soil pH. (**B**) Available phosphorus content. (**C**) Available potassium content. (**D**) Alkaline phosphatase activity. (**E**) Sucrase activity. (**F**) Urease activity. (**G**) Soil organic matter content. (**H**) Total nitrogen content. (**I**) Total phosphorus content. Different lowercase letters indicate significant differences between different treatments (*p* < 0.05).

The effects of various fertilization treatments on the rhizosphere soil factors of *S. miltiorrhiza* were significantly different (Figure 7). Soil pH was higher in all fertilizer treatment groups compared to the control (CK), with significant differences observed in the F3 treatment (Figure 7A). Available phosphorus (AP) and potassium (AK) contents increased under the F1 and F3 treatments (Figure 7B,D), whereas the F2 treatment enhanced both enzyme activity and the soil organic matter (SOM) content (Figure 7G). Notably, AP and sucrase (SC) activity reached their peak levels under the F2 treatment compared to other treatments (Figure 7D,E). The application of foliar fertilizer, both alone and in combination with root fertilizer, significantly increased soil urease activity, while the root fertilizer alone (F1) treatment resulted in a decrease in activity (Figure 7F). Additionally, the F1 and F3 treatments increased the soil total nitrogen (TN) content, whereas the F2 treatment decreased the soil TN content (Figure 7H). The total phosphorus (TP) content in the soil was higher under all fertilizer treatments than under the CK treatment, with significant differences observed in the F2 treatment (Figure 7I).

### 3.8. Correlation Analysis of Endophytic Fungal and Bacterial Communities with Growth Indicators, Medicine Ingredient Contents, and Soil Factors of S. miltiorrhiza

**Figure 8:**
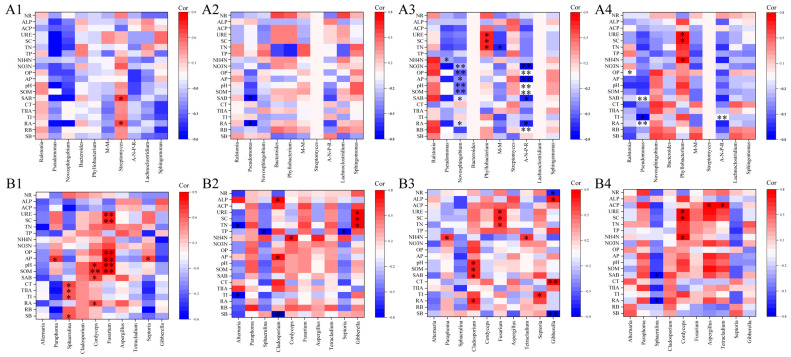
Correlation of endophyte communities with growth indicators, medicine ingredient contents, and soil factors of *S. miltiorrhiza*. (**A**) Endophytic bacteria; (**B**) endophytic fungi. 1–4 indicate the CK, F1, F2, and F3 treatments. SOM: soil organic matter, pH: soil pH, AP: available phosphorus, OP: available potassium, SC: soil sucrase, URE: urease, ACP: acid phosphatase, ALP: alkaline phosphatase, NO3N: nitrate nitrogen, NH4N: ammonium nitrogen, NR: nitrate reductase, TP: total phosphorus, TN: total nitrogen, M-M: methylobacterium–methylorubrum, SB: aboveground biomass, RB: root biomass, RA: rosmarinic acid, SAB: salvianolic acid B, TI: tanshinone I, TIIA: tanshinone IIA, A-N-P-R: allorhizobium–neorhizobium–pararhizobium–rhizobium, CT: cryptotanshinone. The significant statistical results were labeled (* *p  *<  0.05, ** *p*  <  0.01).

Correlation analysis between the top 10 microbial genera and plant growth index, medicinal ingredient contents, and soil factors revealed (Figure 8) that in bacterial communities, *Streptomyces* showed significant positive correlations (*p* < 0.05) with rosmarinic acid and salvianolic acid B under the control treatment. The *Pseudomonas* displayed a significant negative correlation with rosmarinic acid and salvianolic acid B under the F1 and F3 treatments, and *Pseudomonas* showed a negative correlation with tanshinone Ⅰ. In the F2 treatment, A-N-P-R and *Novosphingobium* showed a negative correlation with rosmarinic acid and salvianolic acid B, while *Phyllobacterium* was positively associated with URE, SC, and TN (Figure 8(A3). F2 and F3 treatments enhanced the correlations between endophytic microorganisms and soil characteristics. Notably, the F2 treatment showed more associations, with both A-N-P-R and *Novosphingobium* exhibiting significantly more connections to soil nutrients and enzyme activities than the F3 treatments (Figure 8(A3,A4)).

In fungal communities, *Sphaerulina* was positively correlated with tanshinone IIA, cryptotanshinone, and tanshinone I (*p* < 0.05) under the CK treatment; *Cordyceps* was associated with salvianolic acid B and rosmarinic acid (Figure 8(B1)). In the F1 treatment, *Alternaria* showed significant correlations with tanshinones but negatively with tanshinone I (Figure 8(B2)). *Cladosporium*, *Septoria*, and *Gibberella* were correlated with distinct medicinal compounds under the F2 treatment (Figure 8(B3)). *Sphaerulina* was negatively associated with rosmarinic acid and salvianolic acid B under the F3 treatment (Figure 8(B4)). Notably, a positive correlation was observed between the presence of *Sphaerulina*, *Cordyceps*, *Cladosporium*, and *Septoria* in the fungal community and the content of medicinal constituents across fertilization treatments. In contrast, within the bacterial community, only *Streptomyces* exhibited a positive correlation with both salvinorin B and rosmarinic acid.

### 3.9. Variance Partitioning Analysis

**Figure 9:**
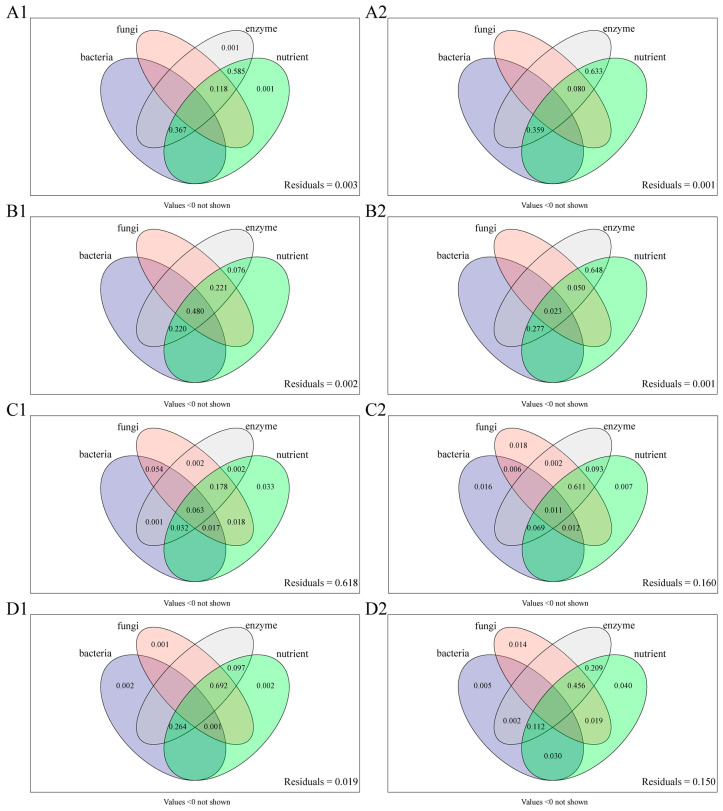
Effects of endophytic microbial diversity indices on growth indices, medicinal ingredient content, and rhizosphere soil factors of *S. miltiorrhiza* under different fertilization treatments. 1 Growth indices; 2 medicine ingredient content. (**A**–**D**) CK, F1, F2, and F3 treatments.

The results of variance partitioning analysis (Figure 9) indicated that endophytic microbial diversity indices did not significantly explain growth or medicinal ingredient content variation in the control (CK) group, with soil nutrients and enzyme activity collectively accounting for only 0.1% of growth index variance, respectively (Figure 9(A1)). The combined effects of endophytic bacterial diversity, fungal diversity, soil nutrients, and enzyme activity accounted for 48.0% of growth index variance and 2.3% of medicinal ingredient content variance under the F1 treatment (Figure 9(B1,B2)). Soil nutrients alone explained 3.3% of growth index variance and 0.7% of medicinal ingredient content variance. In the F2 treatment (Figure 9(C2)), endophytic fungal and bacterial diversity indices explained 1.8% and 1.6% of medicinal ingredient content variance, respectively. The combined explanatory power of all four factors decreased compared to F1. In the F3 treatment, endophytic fungal diversity alone accounted for 1.4% of medicinal ingredient content variance, exceeding the contribution of bacterial diversity (Figure 9(D2)). Collectively, endophytic fungi demonstrated a greater influence than bacteria on *S. miltiorrhiza* quality under both the F2 and F3 treatments. Fungal diversity explained more variance in compound levels than bacterial diversity, especially under foliar fertilizer.

### 3.10. Diversity of Culturable Endophytic Fungi

**Figure 10:**
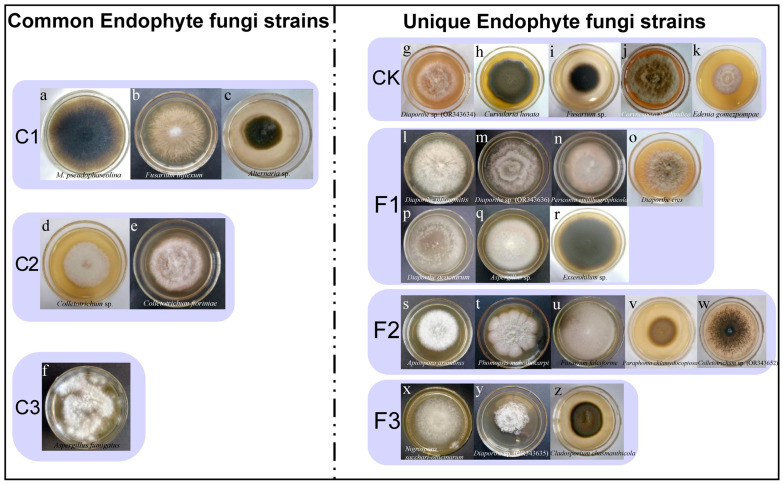
Morphology of endophytic fungal colonies isolated from tissues of *S. miltiorrhiza* with different fertilization treatments. C1 represents common strain species under the CK, F1, F2, and F3 treatments; C2 represents common strain species under the CK and F1 treatments; C3 represents common strain species under the F2 and F3 treatments. The Latin name of the strain is at the bottom of the picture. Letters a–z respectively represent the following fungal strains: *Macrophomina pseudophaseolina*, *Fusarium inflexum*, *Alternaria* sp., *Colletotrichum* sp., *Colletotrichum fioriniae*, *Aspergillus fumigatus*, *Diaporthe* sp. (OR343634), *Curvularia lunata*, *Fusarium* sp., *Corynespora thailandica*, *Edenia gomezpompae*, *Diaporthe phragmitis*, *Diaporthe* sp. (OR343636), *Periconia epilithographicola*, *Diaporthe eres*, *Diaporthe acaciarum*, *Aspergillus* sp., *Exserohilum* sp., *Apiospora arundinis*, *Phomopsis mahothocarpi*, *Fusarium falciforme*, *Paraphoma chlamydocopiosa*, *Colletotrichum* sp. (OR343652), *Nigrospora sacchari officinarum*, *Diaporthe* sp. (OR343635), *Cladosporium chasmanthicola*.

Variance partitioning analysis demonstrated that endophytic fungal diversity contributed more significantly to *S. miltiorrhiza* quality than bacterial diversity (*p* < 0.05). In this study, 26 strains of endophytic fungi belonging to 16 genera were successfully isolated from tissues of different ecological niches of *S. miltiorrhiza* under different fertilization treatments (Figure S2A–D). The isolated endophytic fungi represented 16 genera: *Alternaria*, *Apiospora*, *Aspergillus*, *Cladosporium*, *Colletotrichum*, *Corynespora*, *Curvularia*, *Diaporthe*, *Edenia*, *Exserohilum*, *Fusarium*, *Macrophomina*, *Nigrospora*, *Paraphoma*, *Periconia*, and *Phomopsis*. Among these endophytic fungal strains, five unique strains (*Curvularia lunata*, *Corynespora thailandica*, *Diaporthe* sp. (OR343634), *Edenia gomezpompae*, and *Fusarium* sp.) were isolated from *S. miltiorrhiza* under the CK treatment (Figure 10g–k). Seven unique strains (*Diaporthe acaciarum*, *Diaporthe eres*, *Diaporthe phragmitis*, *Diaporthe* sp. (OR343636), *Periconia epilithographicola*, *Exserohilum* sp., and *Aspergillus* sp.) were isolated from *S. miltiorrhiza* under the F1 treatment (Figure 10l–r). The F2 and F3 treatments of *S. miltiorrhiza* isolated five (*Apiospora arundinis*, *Colletotrichum* sp. (OR343652), *Fusarium falciforme*, *Phomopsis mahothocarpi*, and *Paraphoma chlamydocopiosa*) and three (*Cladosporium chasmanthicola*, *Diaporthe* sp. (OR343635), and *Nigrospora sacchari officinarum*) unique strains (Figure 10s–z). Three shared strains were isolated under four fertilization treatments, including *Alternaria* sp., *Fusarium inflexum*, and *Macrophomina pseudophaseolina* (Figure 10a–c). *Colletotrichum* sp. (OR343631) and *Colletotrichum fioriniae* were the common strains for the CK and F1 treatments (Figure 10d,e). In addition, the common strain *Aspergillus fumigatus* was isolated under the F2 and F3 treatments (Figure 10f).

## 4. Discussion

### 4.1. Effects of Different Fertilization Treatments on Endophytic Bacterial Communities of S. miltiorrhiza

Previous studies indicate that Proteobacteria, which includes numerous pathogenic species, represents the most prevalent endophytic bacterial phylum in plants [45]. In our study, Proteobacteria predominated in Mimosa tissue endophytes, with fertilization treatments significantly altering their relative abundance. Compared to the control, the F1 and F2 treatments increased Ascomycota proportions more substantially than F3, suggesting the need for optimized fertilizer combination ratios. At the genus level, *Ralstonia* showed the highest relative abundance across treatments, particularly in F2. Macroelement foliar fertilizer affected soil fungi by directly affecting the growth parameters and macroelement content of the plant or by directly affecting soil properties [46]. *Ralstonia* readily invades the root system and colonizes plant xylem [47]; it is also a known pathogen. Notably, the abundance of *Ralstonia* decreased under the F3 treatment. Furthermore, a significant correlation between *Ralstonia* and available potassium was observed exclusively in the F3 treatment. Fertilizer application strengthened microbe–soil interactions, with *Novosphingobium* showing significantly stronger correlations with soil nutrients and enzyme activities under the F3 treatment compared to F1 and F2. Fertilizer-enhanced soil nutrients create favorable conditions for microbial proliferation. *Novosphingobium* strains utilize aromatic carbon sources and can be engineered for bioproduct synthesis [48]. Research demonstrates that *Novosphingobium* sp. enhances citrus salt stress tolerance while promoting growth when used as inoculants [49]. Meanwhile, *Pseudomonas* represented a significant proportion across all treatments. As previously demonstrated, *Pseudomonas* species are widely distributed in plant root systems and exhibit biological control potential [50]. In our study, *Pseudomonas* abundance showed significant negative correlations with both root biomass and the rosmarinic acid content, specifically under the F2 treatment. These findings demonstrate differential effects of fertilizer treatments on endophytic bacterial abundance, with endophytic bacterial communities showing a correlation with soil physicochemical properties, plant growth performance, and the accumulation of medicinal ingredient contents.

In this experiment, the Shannon index of endophytic bacteria decreased across all three fertilization treatments, with the most pronounced reduction observed under the F2 treatment. These results align with previous reports demonstrating that fertilizer application can alter bacterial abundance dynamics [51]. OTU analysis indicated that bacterial community richness was significantly higher in the F3 treatment compared to the others, likely due to the combined application of foliar and root fertilizers. Functional prediction analysis indicated relatively stable functional profiles of the bacterial communities across treatments, though a considerable proportion of functions remained unclassified.

### 4.2. Effects of Different Fertilization Treatments on Endophytic Fungal Communities of S. miltiorrhiza

The diversity of soil fungal communities was consistently higher than that of bacterial communities across all treatments. Notably, the unfertilized control (CK) showed significant differences from fertilized treatments, indicating that fertilizer application directly influences fungal diversity [52]. Ascomycota emerged as the dominant phylum in *S. miltiorrhiza* tissues across all treatments, consistent with previous findings [53]. At the genus level, *Alternaria* predominated in the fungal communities, although its relative abundance varied substantially among treatments: CK (44.72%), F1 (23.25%), F2 (28.61%), and F3 (28.60%). Notably, a negative correlation between *Alternaria* abundance and both tanshinone I content and total nitrogen was observed exclusively in the F1 treatment. As a genus containing numerous globally significant crop pathogens, *Alternaria* species cause diseases including pistachio wilt [54], tomato early blight [55], and coriander wilt [56]. Similarly, *Aspergillus*, an economically important post-harvest pathogen [57], showed abundance variations in response to different fertilizer treatment. However, no significant correlations were detected between *Aspergillus* abundance and either soil nutrients or plant growth parameters. Interestingly, both *Aspergillus* and *Paraphoma* demonstrate antagonistic activity against plant pathogens [58]. *Paraphoma* abundance increased specifically under the F2 (foliar spray) treatment while decreasing under the F1 and F3 treatments, potentially reflecting its documented efficacy against leaf brown spot disease. The exclusive detection of Mucoromycota in the F3 treatment (combined root and foliar fertilization) may reflect its ecological preference for decomposing wet organic matter, suggesting treatment-specific microbial community shifts. The studies have shown that applying root fertilizer increased crop output considerably [59]. In this study, root fertilizer application significantly increased root biomass, whereas foliar fertilization enhanced aboveground biomass. Conversely, the combined fertilization did not enhance growth, possibly due to over-application or nutrient imbalance.

Co-occurrence network analysis revealed potential microbial interactions [60]. Among all fertilization treatments, foliar application resulted in the highest modularity index for endophytic fungi, indicating the most stable community structure. Such stable symbiotic networks may enhance nutrient partitioning and ecosystem stability [61]. Functional prediction of the endophytic fungal communities showed that pathotroph–saprotroph–symbiotroph trophic modes predominated across all treatments, though their relative abundances decreased following fertilizer application compared to the control. The potential functions of endophytes varied across treatments, possibly due to differences in the plant’s survival environment. OTU analysis revealed that the F1 treatment contained more unique OTUs than the F2 or F3 treatments. This pattern likely occurred because the root fertilizer enriched soil nutrients, thereby promoting microbial survival and attracting beneficial microorganisms [62].

### 4.3. Effect of Fertilization Treatments on Quality and Soil Factors of S. miltiorrhiza

Studies have shown that applying root fertilizer increased crop output considerably [59]. In this investigation, root fertilizer treatment considerably increased root biomass, whereas foliar fertilizer treatment raised biomass and co-treatment of root and foliar fertilizers decreased biomass. This suggests that foliar fertilizers can increase the biomass of *S. miltiorrhiza*, which is consistent with the findings of Geneva et al. [63]. The combination of root and foliar fertilizers can reduce the burden of root fertilizers on the soil, but in this experiment, the co-treatment group of root and foliar fertilizers did not significantly promote the growth of *S. miltiorrhiza*, which could be attributed to the irrational fertilizer ratio and over-application. Notably, all three fertilization treatments significantly increased the accumulation of salvianolic acid B and tanshinone IIA, whereas both foliar and root fertilizer treatments increased the accumulation of tanshinone I and cryptotanshinone, but the combination of root and foliar fertilizers inhibited the accumulation of tanshinone I and cryptotanshinone. Li et al. [64] discovered that, as compared to organic and chemical fertilizer ratios of 3:7 and 5:5, the quality and yield of the medicinal plant *Euryales Semen* were greatly improved at 7:3. As a result, the ratio of root–foliar fertilizer needs to be further explored to enhance the biomass and medicinal ingredient of *S. miltiorrhiza*.

Plant growth, development, and defense are heavily dependent on soil nutrients [65]. Fertilizer application has been found to promote plant growth by adding additional soil nutrients [66]. However, a lack of mineral nutrients such as nitrogen (N), phosphorus (P), and potassium (K) can limit crop growth and production [67]. The soil content of fast-acting N, P, and K was found to increase under fertilizer treatments in the present study, which is consistent with previous research [68]. It is noteworthy that N, P, and K contents were significantly increased under the F1 and F3 treatments, which may be attributed to the fact that the fertilizers were applied to the soil in a root manner and stored more in the soil. Meanwhile, foliar spraying is more readily absorbed by the plant, and the remaining portion is washed into the soil by rainwater [69]. Soil enzyme activities are now widely used as important indicators of soil quality and biological activity in soil [70]. Among them, urease and alkaline phosphatase are the more sensitive enzymes to soil fertility. ALP activity in soil under different fertilization treatments was significantly higher than that in CK, which was consistent with the results of Wang et al. [58]. In addition, URE activity was significantly lower than CK in the F1 treatment group, while URE activity was highest under the F3 treatment, which might be the effect of the co-application of two fertilizers. Jabborova et al. [71] found that the NPK + BZnFe fertilizer combination increased urease and alkaline phosphatase activities in soil as well as the nutrient content in Curcuma longa rhizomes in a field experiment. Sucrase is involved in the conversion of carbohydrate production in the soil and can hydrolyze soil organic matter into glucose and sucrose, which are easily available for plant uptake and utilization and are required for the survival of soil microorganisms [72]. The results of the study showed that the F2 and F3 treatments significantly increased soil SC activity as compared to the control. Bai et al. [73] found that more rationed phosphorus fertilizers had the greatest effect on soil sucrase activity through different combinations of N, P, and K fertilizer treatments; in addition, plants exhibited the highest plant height at a P fertilizer ratio of 180 kg/hm^2^. Soil organic matter (SOM) provides both energy and nutrients to soil-associated microorganisms and is a product of soil microorganisms and plant spoilage bodies [74]. The F2 treatment exhibited a substantially higher SOM content than the other groups, which is consistent with the findings of the previous study [75]. In addition, the F1 and F3 treatments also increased the SOM content. Zhou et al. [76] found that NPK fertilization affected the changes in SOM content more than when N fertilization was applied alone.

### 4.4. Effects of Different Fertilization Treatments on Endophytic Microbial Communities of S. miltiorrhiza

High-throughput technologies have been demonstrated to play a pivotal role in identifying functional microbial strains and characterizing microbial diversity [77]. Specifically, Lei et al. [78] revealed that fertilizer application significantly alters the endophytic microbial diversity in tea plants through high-throughput sequencing analysis. Similarly, Gong et al. [15] employed high-throughput sequencing to demonstrate fertilizer-induced changes in the epiphytic microbial communities of *S. miltiorrhiza*. Our experimental results further confirmed that different fertilization regimens distinctly influenced the composition of endophytic microbial communities in *S. miltiorrhiza*.

Beyond community characterization, high-throughput technologies enable simultaneous detection of phytopathogenic microorganisms and identification of beneficial microbes capable of pathogen suppression and plant growth promotion [79]. Variance partitioning analysis (VPA) revealed that endophytic fungal diversity indices under the F2 and F3 treatments exhibited stronger correlations with medicinal compound contents in *S. miltiorrhiza* compared to bacterial diversity indices. Previous studies have demonstrated that fertilization increases both the α-diversity and abundance of endophytic fungal communities [80]. It has been shown that endophytic fungi enhance plant growth and medicine quality through multiple mechanisms, including phytostimulation, biocontrol, and biofertilization [81]. For example, beneficial microorganisms in the rhizosphere of *Hypericum perforatum* can stimulate secondary metabolism, elevating the content of chrysin and pseudochrysin [82]. Therefore, in this study, we isolated culturable endophytes from *S. miltiorrhiza* tissues under fertilization treatments to provide microbial resources for subsequent functional validation.

Numerous studies have successfully isolated and characterized growth-promoting endophytic fungi from medicinal plants [81,83]. In the current study, we complemented high-throughput analyses with traditional culture-based methods to investigate culturable endophytic fungal communities across fertilization treatments. From *S. miltiorrhiza* tissues, we isolated 26 fungal strains representing 16 genera. Notably, the F1 treatment yielded higher numbers of unique strains than F2 and F3, potentially attributable to root fertilizer-induced improvements in rhizosphere nutrient availability [84]. The predominant genus among F1 isolates was Diaporthe, consistent with its recognition as one of the most ubiquitous endophytic fungal genera with broad host ranges worldwide [85].

Three fungal species—*Alternaria* sp., *Fusarium inflexum*, and *Macrophomina pseudophaseolina*—were shared across all fertilization treatments. While *Alternaria* exhibits remarkable metabolic adaptability to diverse hosts and environments [86], and *M. pseudophaseolina* is a known causative agent of charcoal rot in oilseed crops [87], recent evidence suggests potential beneficial roles. Xie et al. [88] reported drought tolerance enhancement in *Astragalus* through *M*. *pseudophaseolina* inoculation, and certain *Fusarium* strains (e.g., HPF-1) demonstrate biocontrol activity against pathogenic *Fusarium* species in orchids [89]. These findings underscore the potential for reintroducing cultured endophytes to study their effects on host plant productivity, growth enhancement, and carbon metabolism efficiency.

## 5. Conclusions

This study investigated the effects of fertilization on both the quality of *S. miltiorrhiza* and the composition of its endophytic microbial communities. The results demonstrated that the F1 treatment significantly increased both the root biomass and the content of tanshinone and salvianolic acid B; the F2 treatment markedly enhanced the aboveground biomass. Fertilization significantly altered the community structure of endophytic microorganisms in *S. miltiorrhiza*, with Ascomycota (fungi) and Proteobacteria (bacteria) emerging as the dominant phyla. Notably, the F3 treatment resulted in significantly greater Proteobacteria abundance compared to other treatments. In addition, the F2 treatment revealed significant positive correlations between *Septoria* and tanshinone I and between *Gibberella* and cryptotanshinone. Through cultivation-based isolation, we identified 26 culturable endophytic fungal species representing 16 genera across all treatments. Notably, the F1 treatment yielded the highest number of unique strains. This study highlights fungal community shifts as key drivers of metabolite variation in *S. miltiorrhiza*, with implications for microbial-based cultivation strategies.

## Data Availability

The original contributions presented in this study are included in the article/Supplementary Materials, and further inquiries can be directed to the corresponding authors.

## Supplementary Materials

The following supporting information can be downloaded at: https://www.mdpi.com/article/10.3390/microorganisms13061429/s1, Table S1: Mobile phase conditions for fat-soluble components. Figure S1: Sparse curve based on Good’s coverage of *Salvia miltiorrhiza* endophytic bacteria (A) and fungi (B) OTUs. Figure S2. Maximum likelihood tree for different fertilization treatments based on rDNA ITS sequences of endophytic fungi of *S. miltiorrhiza*. (A) indicated CK treatment. (B) indicated root fertilizer treatment (F1). (C) indicated foliar fertilizer treatment (F2). (D) indicated root fertilizer + foliar fertilizer treatment (F3).
