# Supplementary material for: Response of Endophytic Microbial Communities and Quality of Salvia miltiorrhiza to Fertilization Treatments
Source: Microorganisms. 2025 Jun 19;13(6):1429. doi: 10.3390/microorganisms13061429 (PMC12196437; doi:10.3390/microorganisms13061429)
Supplement: Supplementary file 1 [file microorganisms-13-01429-s001.zip › Supplementary Material.pdf]

Supplementary Material

1 Supplementary Figures and Tables

1.1 Supplementary Tables

Table S1 Mobile phase conditions for fat-soluble components

Time (min)	mobile phases A (%)	mobile phases B (%)
0~6	61	39
6~20	61-90	39-10
20~20.5	90-61	10-39
20.5~25	61	39

1.2 Supplementary Figures

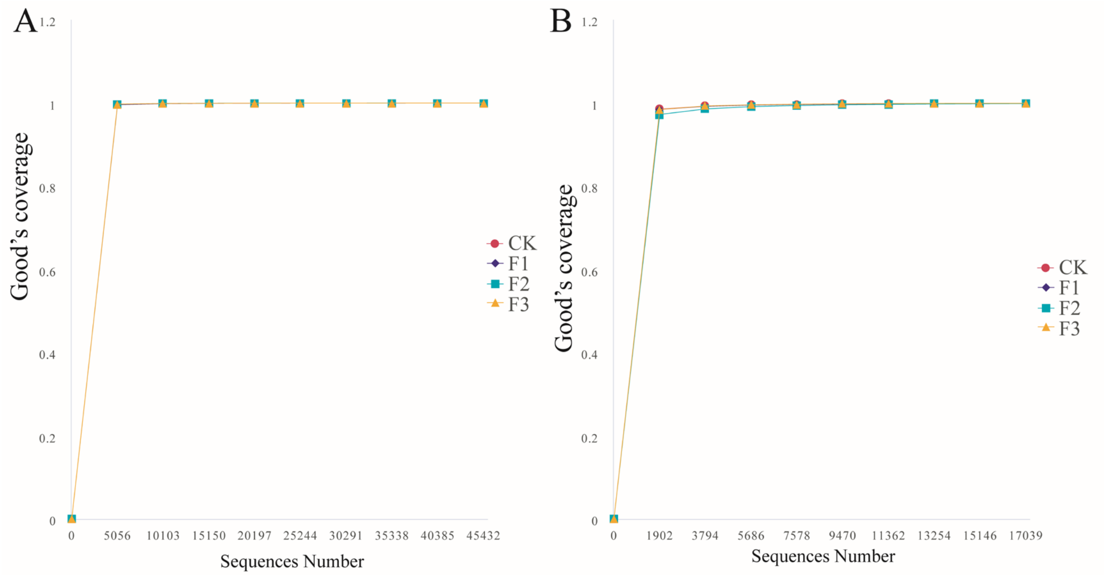

Figure S1. Sparse curve based on Good's coverage of *Salvia miltiorrhiza* endophytic bacteria (A) and fungi (B) OTUs

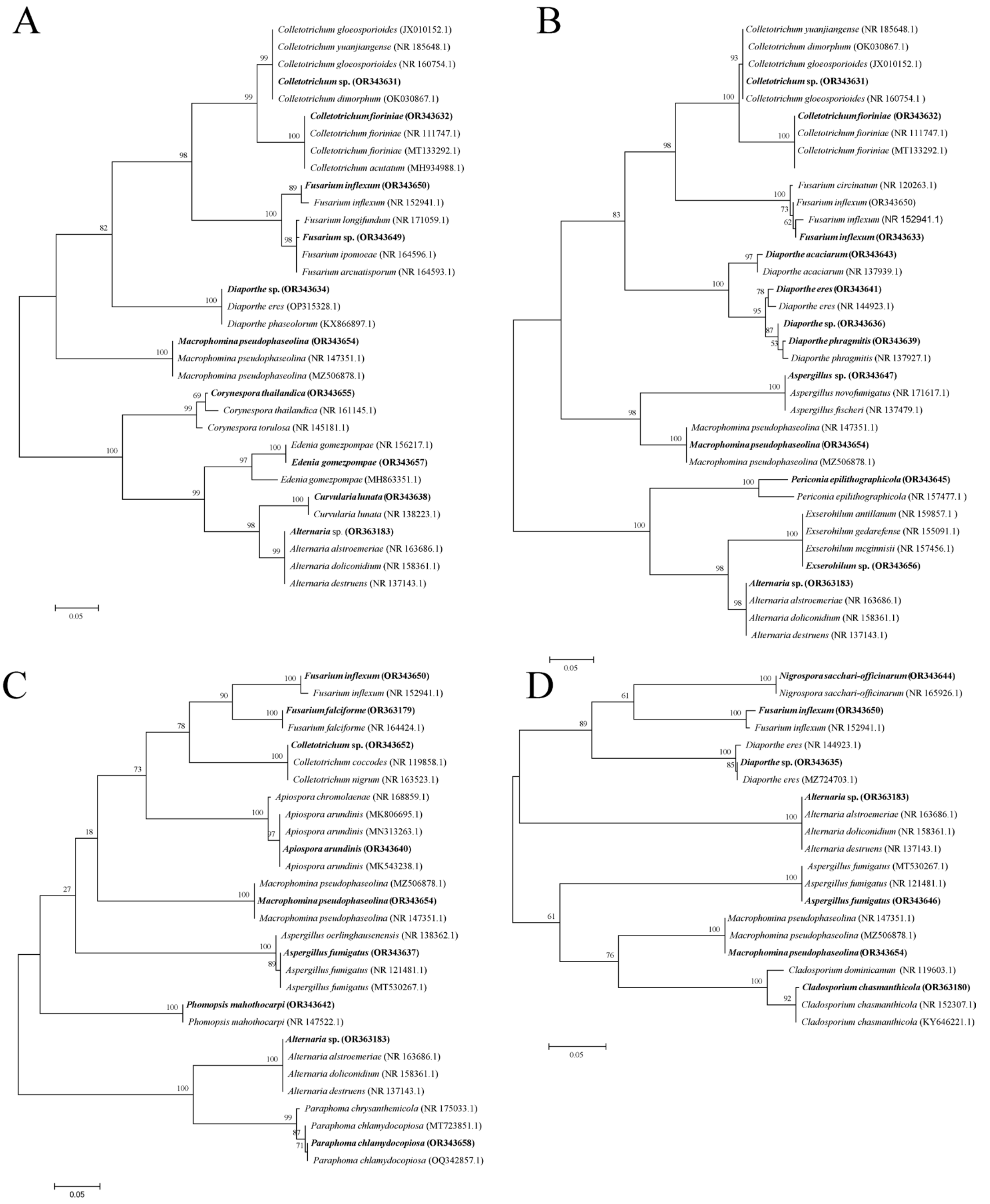

Figure S2. Maximum likelihood tree for different fertilization treatmentsbased on rDNA ITS sequences of endophytic fungi of *S. miltiorrhiza*. (A) indicated CK treatment. (B) indicated root fertilizer treatment (F1). (C) indicated foliar fertilizer treatment (F2). (D) indicated root fertilizer + foliar fertilizer treatment (F3).
